# Real-life evaluation of BioFire^®^ BCID2 and Vitek^®^ REVEAL^TM^ for Gram-negative bloodstream infections in critical care

**DOI:** 10.1371/journal.pone.0356199

**Published:** 2026-09-10

**Authors:** Massinissa Benyahia, Claire Roger, Adeline Boutet-Dubois, Florian Salipante, Louis Oudiane, Matthieu Curtil Dit Galin, Rim Nsiri, Léo Brochon, Jean-Philippe Lavigne, Alix Pantel

**Affiliations:** 1 Department of Microbiology and Hospital Hygiene, Nîmes University Hospital, Nîmes, France; 2 Division of Anesthesia and Critical Care, UR-UM103 IMAGINE, University of Montpellier, Pain and Emergency Medicine, Nîmes University Hospital, Nîmes, France; 3 Department of Biostatistics, Epidemiology, Public Health, and Innovation in Methodology (BESPIM), Nîmes University Hospital, Nîmes, France; 4 VBIC, INSERM U1047, University of Montpellier, Nîmes, France; Children’s National Hospital, George Washington University, UNITED STATES OF AMERICA

## Abstract

Timely management of sepsis relies on rapid diagnosis of bloodstream infections (BSIs), including early pathogen identification and antimicrobial susceptibility testing (AST). The Vitek^®^ REVEAL^TM^ is an innovative rapid AST platform that detects volatile organic compounds emitted by Gram-negative bacilli (GNB) directly from positive blood cultures. This study assessed the analytical performance and clinical contribution of the BioFire^®^ BCID2 molecular panel and the Vitek^®^ REVEAL^TM^ system in the diagnosis of GNB-BSI among intensive care unit (ICU) patients. We analyzed the prospective phase of the BacteREVEAL study, conducted between March and October 2023 at Nîmes University Hospital. Results from BioFire^®^ BCID2 and Vitek^®^ REVEAL^TM^ were compared to standard-of-care methods, including MALDI-TOF identification and direct disk diffusion AST. Performance metrics for Vitek^®^ REVEAL^TM^ were evaluated according to ISO 20776–2:2007 standards. Among 94 ICU patients with a first episode of GNB-BSI, 50 monomicrobial cases were included. *Escherichia coli* was the most frequently identified species (n = 30, 60%), followed by *Pseudomonas aeruginosa* (n = 7, 14%). BioFire^®^ BCID2 provided accurate identification for all isolates. The innovative tools reduced median turnaround times for identification by 19.0 h and for AST by 14.9 h. The mean time to result for Vitek^®^ REVEAL^TM^ was 5.7 h. Overall categorical agreement with standard AST was 97.7%, with 8 very major errors, 8 major errors, and 2 minor errors. These findings support the integration of BioFire^®^ BCID2 and Vitek^®^ REVEAL^TM^ into routine workflows to accelerate GNB-BSI diagnosis and improve sepsis management.

## Introduction

Bloodstream infections (BSIs) represent a growing public health concern due to their association with significant morbidity and mortality [1–3]. In intensive care unit (ICU) settings, BSIs rank as the third most common infection, with Gram-negative bacilli (GNB) being the predominant pathogens [4–6].

Timely initiation of effective and appropriate antimicrobial therapy is critical to improve patient outcomes [7,8]. However, the increasing prevalence of antibiotic resistance among GNB isolates [9–11] complicates the selection of initial empiric therapy, often leading to delays in appropriate treatment, prolonged hospital stays, and increased mortality [12,13].

To address this challenge, rapid molecular diagnostic tools have emerged as valuable assets for early pathogen identification [14–16]. Among these, the BioFire^®^ FilmArray^®^ Blood Culture Identification 2 (BCID2) panel (bioMérieux, Marcy l’Etoile, France) is a highly multiplexed PCR assay capable of detecting a broad range of bacterial and fungal pathogens, along with key resistance genes [16,17]. Its user-friendly design and rapid turnaround time makes it an interesting tool for accelerating the diagnostic process. However, molecular tests are inherently limited in the ability to predict phenotypic antimicrobial susceptibility, due to the complexity and diversity of resistance mechanisms and the restricted scope of resistance gene panels. To complement molecular diagnostics, rapid antimicrobial susceptibility testing (AST) performed directly on positive blood cultures offers a phenotypic profile of antimicrobial resistance. This enables both escalation in the presence of resistance and de-escalation to preserve broad-spectrum agents when susceptibility is confirmed [18].

Several rapid AST devices designed for direct use on positive BCs had been developed and evaluated in the past years. These tools rely on a wide variety of technologies such as morphokinetic cellular analysis, broth-microdilution with time-lapse microscopy or flow cytometry [18].

The Vitek^®^ REVEAL^TM^ system (bioMérieux) introduces an innovative approach by detecting volatile organic compounds (VOCs) emitted by growing bacteria exposed to various concentrations of antibiotics, using colorimetric sensor technology. Since each bacterial species produces a unique VOC signature [19], this method enables rapid phenotypic AST results and determination of minimum inhibitory concentrations (MICs).

Between 2021 and 2023, we conducted a quasi-experimental pre/post interventional study at Nîmes University Hospital (BacteREVEAL study, ClinicalTrials.gov identifier: NCT05741424), evaluating the clinical impact of implementing these innovative tools in ICU patients with GNB-BSI [20].

The objectives of the present work were *i)* to evaluate the analytical performance of the BioFire^®^ BCID2 panel and the Vitek^®^ REVEAL^TM^ system during the post-intervention phase of the BacteREVEAL study, and *ii)* to assess the contribution of these technologies in improving the diagnosis of GNB-BSI.

## Materials and methods

### Study setting and population

The BacteREVEAL study was a pre/post interventional investigation conducted at the Nîmes University Hospital, a 2000-bed tertiary care center in southern France, comprising three ICUs with a total of 50 beds. This work focuses on the post-intervention phase, which included adult ICU patients with a first episode of monomicrobial GNB-BSI between 1 March and 20 October 2023. The study was approved by a French Ethics Committee (Comité de Protection des Personnes Ouest I, 2022-A02294-39), and informed consent was obtained from all participants. Patients were eligible if blood cultures (BCs) were collected either in the ICU or in another department, provided they were admitted to the ICU at the time of BC positivity. Exclusion criteria included moribund status, a second episode of bacteremia or lack of informed consent.

### Diagnosis of bloodstream infections

For each patient, four to six bottles of BacT/Alert^®^ FA and FN PLUS (bioMérieux) BCs were collected and sent to the clinical microbiology laboratory, which operates 24/7 and is located in the same building as the ICUs. BCs were continuously processed and incubated for up to five days using the BacT/Alert^®^ VIRTUO^TM^ automated system (bioMérieux). Positive BCs were handled from 8:00 AM to midnight using both conventional and innovative diagnostic methods.

### Conventional workflow

Gram staining was performed on all positive BCs. Direct AST was conducted on monomicrobial GNB isolates using the disk diffusion method, following the guidelines of the Antibiogram Committee of the French Society of Microbiology (CA-SFM) [21]. Briefly, 200 µL of positive BC was diluted in 10 mL of 0.9% NaCl, and the suspension was used to inoculate Mueller-Hinton agar plates. Antibiotic disks were applied, and plates were incubated at 35°C for 16–24 h. The following morning, bacterial identification was performed using by MALDI-TOF mass spectrometry (Vitek^®^ MS, bioMérieux) on subcultures. Inhibition zone diameters were measured using the SIRweb software (i2a, Montpellier, France), and AST results were interpreted according to CA-SFM/EUCAST (European Committee on Antimicrobial Susceptibility Testing) 2023 breakpoints [21,22]. For antibiotics not included in the routine panel, MICs were determined using ETEST^®^ strips (bioMérieux): tigecycline for *Escherichia coli*, and ceftolozane-tazobactam and ceftazidime-avibactam for *Pseudomonas aeruginosa*.

### Innovative diagnosis workflow

Upon Gram staining confirmation of GNB, rapid identification was performed directly on the positive BC using the BioFire^®^ FilmArray BCID2 panel (bioMérieux) according to the manufacturer’s instructions.

Simultaneously, rapid AST was initiated using the Vitek^®^ REVEAL^TM^ system, without waiting for identification results and within 16 h of BC positivity, as per manufacturer’s recommendations. A 25 µL aliquot of positive BC was diluted in 25 mL of Pluronic water (Beckman Coulter, Villepinte, France), and 115 µL of this suspension was inoculated into each well of the GN01 antibiotic plate (bioMérieux) using the Renok inoculator (Beckman Coulter). Plates were sealed with the Vitek^®^ REVEAL^TM^ sensor and incubated in the Vitek^®^ REVEAL^TM^ instrument. Once available, BCID2 identification data were entered into the system to enable result interpretation. The antimicrobials tested on the GN01 plate are listed in **S1 Table**.

Only positive BCs yielding GNB species validated for use with the Vitek^®^ REVEAL^TM^ system were included: *E. coli, Klebsiella pneumoniae, Klebsiella aerogenes, Klebsiella oxytoca, Enterobacter cloacae* complex, *Citrobacter freundii, Citrobacter koseri, Proteus mirabilis, P. aeruginosa* and *Acinetobacter baumannii.* Quality control was performed monthly using ATCC strains: *E. coli* ATCC 25922, *P. aeruginosa* ATCC 27853, and *K. pneumoniae* ATCC 700603.

### Discrepancies analysis

In cases of discordance between disk diffusion and Vitek^®^ REVEAL^TM^ AST results, MICs were determined on subcultures using the following methods: UMIC^®^ broth-microdilution (Bruker, Wissembourg, France) for piperacillin-tazobactam; ETEST^®^ strips for piperacillin, cefotaxime, aztreonam, ertapenem, meropenem, and ciprofloxacin; and Sensititre^TM^ EUMDRXXF plates (Thermo Fisher Scientific, Waltham, MA, USA) for tobramycin.

### Comparative analysis

All Vitek^®^ REVEAL^TM^ results for the 50 GNB isolates were included in the calculation of average time to result (TTR), including those involving intrinsic resistance (e.g., piperacillin for *K. pneumoniae* and *K. oxytoca*). Categorical agreement (CA) percentages were calculated according to ISO 20776–2:2007 standards, with an acceptability threshold of ≥90% [23]. Rates of very major errors (VME), major errors (ME), and minor errors (mE) were also determined per ISO 20776–2:2007 guidelines [23]. Results from Extended Spectrum β-lactamase (ESBL) screening, invalid tests, and intrinsic resistances were excluded from clinical performances calculations. Turnaround times between conventional and innovative methods were compared using the Wilcoxon rank sum test. A p-value ≤ 0.05 was considered statistically significant. Statistical analyses were performed using R v4.3.2 (R Foundation for Statistical Computing).

### Characterization of ESBL-encoding genes

For the confirmed ESBL-producing strains, whole genome sequencing was performed using Illumina MiSeq^®^ system (Illumina, San Diego, CA, United States). The EPISEQ^®^ CS v2.0 (bioMérieux) software was used for bioinformatical analysis and resistome characterization.

## Results

### Study population and diagnostic overview

Among 94 ICU patients with a first episode of GNB-BSI between March and October 2023, 50 patients with monomicrobial GNB bacteremia were included in the study (**Table 1**). The median age was 71 years. The most common comorbidities were arterial hypertension (46%) and diabetes mellitus (32%). Urinary tract infections were the main sources of the BSIs (38%), followed by intra-abdominal infections (34%) (**Table 2**). The BioFire^®^ BCID2 panel showed 100% concordance with MALDI-TOF identification across all 50 cases. The most frequently identified species was *E. coli,* accounting for 30 episodes (60%). No resistance genes were detected by the BioFire^®^ BCID2 assay. Eight isolates (16%) were classified as multidrug-resistant (**Table 2**).

**Table 1:** Demographic and clinical characteristics of ICU patients with monomicrobial Gram-negative bloodstream infections included in the study.

Patients characteristics	Total (n = 50)
**Demographics**	
Age (years)^a^	71 (62-76)
Sex ratio (♂/♀)	34/16
BMI (kg/m^2^)^a^	26 (23-30)
**SOFA score** ^**a**^	7.0 (4.0-9.0)
**Comorbidities** ^b^	
Arterial hypertension	23 (46%)
Diabetes mellitus	16 (32%)
Chronic renal failure	8 (16%)
COPD	7 (14%)
Immunodepression	7 (14%)
Coronaropathy	3 (6%)
Hepatic cirrhosis Child A-B	1 (2%)

^a^Data are expressed as median (interquartile range).

^b^Data are expressed as number of cases (proportion).

BMI, Body mass index; SOFA, Sequential organ failure assessment; COPD, Chronic obstructive pulmonary disease.

**Table 2:** Microbiological characteristics of the monomicrobial Gram-negative bacilli bloodstream infections included in the study.

Source of bloodstream infection	Species							Total
	*Escherichia coli* (n = 30)	*Pseudomonas aeruginosa* (n = 7)	*Enterobacter cloacae* complex (n = 4)	*Klebsiella pneumoniae* (n = 3)	*Klebsiella aerogenes* (n = 2)	*Klebsiella oxytoca* (n = 2)	*Proteus mirabilis* (n = 2)	
Urinary	14	2	1	0	0	1	1	19 (38%)
Digestive	11	2	1	1	1	0	1	17 (34%)
Pulmonary	3	1	0	1	0	1	0	6 (12%)
Intravascular catheter	0	0	1	0	1	0	0	2 (4%)
Skin and soft tissue infections	0	1	0	0	0	0	0	1 (2%)
Other	2	1	1	1	0	0	0	5 (10%)
**Multidrug-resistant isolates**								
ESBL	1	0	0	1	0	0	0	2
Overproduced AmpC	2	1	0	0	0	0	0	3
ESAC	0	0	1	0	1	0	0	2
Overproduced OXY-1	NA	NA	NA	NA	NA	1	NA	1

ESBL, Extended-spectrum beta-lactamase; ESAC, Extended-spectrum AmpC; NA, Not Applicable.

Forty-four BSIs were excluded despite initial Gram stain detection of GNB (S2 Table). The most common exclusion reason was identification of a species not included in the Vitek® REVEAL^TM^ panel (18 cases, 40.9%) (S3 Table). The second most frequent reason was polymicrobial infection (12 cases, 27.3%), detected either by BioFire^®^ BCID2 and subcultures (n = 6) or by subcultures only (n = 6).

### Turnaround times between BC reception and positivity

**Fig 1:**
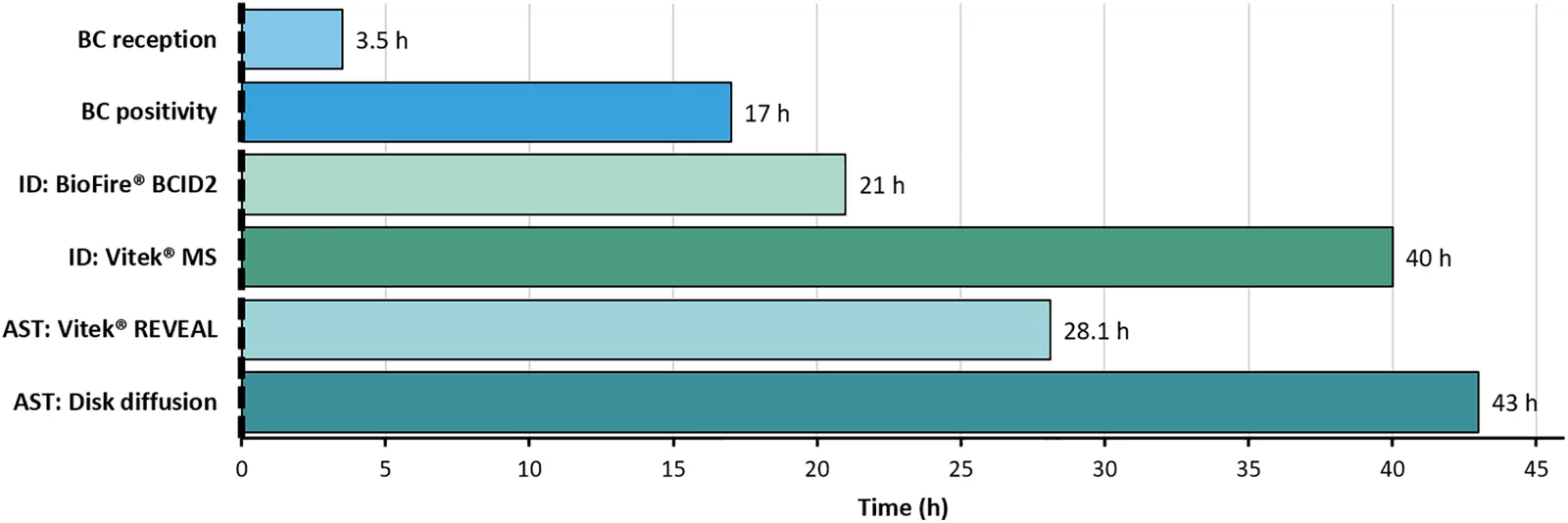
Median turnaround times for each diagnostic step following blood culture collection.

Fig 1 illustrates the median turnaround times for each diagnostic step following BC collection. The median time from collection to laboratory reception was 3.5 h (interquartile range [IQR] 2.7–5.1). Median time to BC positivity was 17 h post-collection (IQR 13–20), corresponding to 11 h post-incubation (IQR 9–15).

### Turnaround times for bacterial identification and AST results delivery

The BioFire^®^ BCID2 panel significantly reduced the time to identification compared to Vitek^®^ MS (4.0 h [IQR 2.5–8.5] *vs* 23 h, [IQR 16–30]; p < 0.001). Vitek^®^ REVEAL^TM^ provided AST results significantly faster than conventional disk diffusion (11.1 h [IQR 9.5–15.5] *vs* 26 h [IQR 18–31]; p < 0.001).

As shown in **Table 3**, turnaround times were significantly shorter when BCs became positive between 8:00 AM and midnight compared to overnight (midnight – 8:00 AM): 2.9 h for identification and 10.3 h for RAST *vs* 8.5 h for identification and 15.5 h for RAST (p < 0.001).

**Table 3:** Median turnaround times for Biofire^®^ BCID2 and Vitek^®^ REVEAL^TM^ results according to the time of blood culture positivity.

Result	Overall (n = 50)	8:00 AM to midnight positive BCs (n = 29)	Midnight to 8:00 AM positive BCs (n = 21)	p-value^a^
BioFire^®^ BCID2	4.0 (2.5 - 8.5)	2.9 (2.0 - 3.4)	8.5 (8.0 - 10.2)	< 0.001
Vitek^®^ REVEAL^TM^	11.1 (9.5 - 15.5)	10.3 (8.8 - 10.3)	15.5 (13.6 - 16.7)	< 0.001

Data are expressed as median (IQR). BC, Blood culture.

^a^Wilcoxon signed rank sum test.

### Time to results of Vitek^®^ REVEAL^TM^

**Fig 2:**
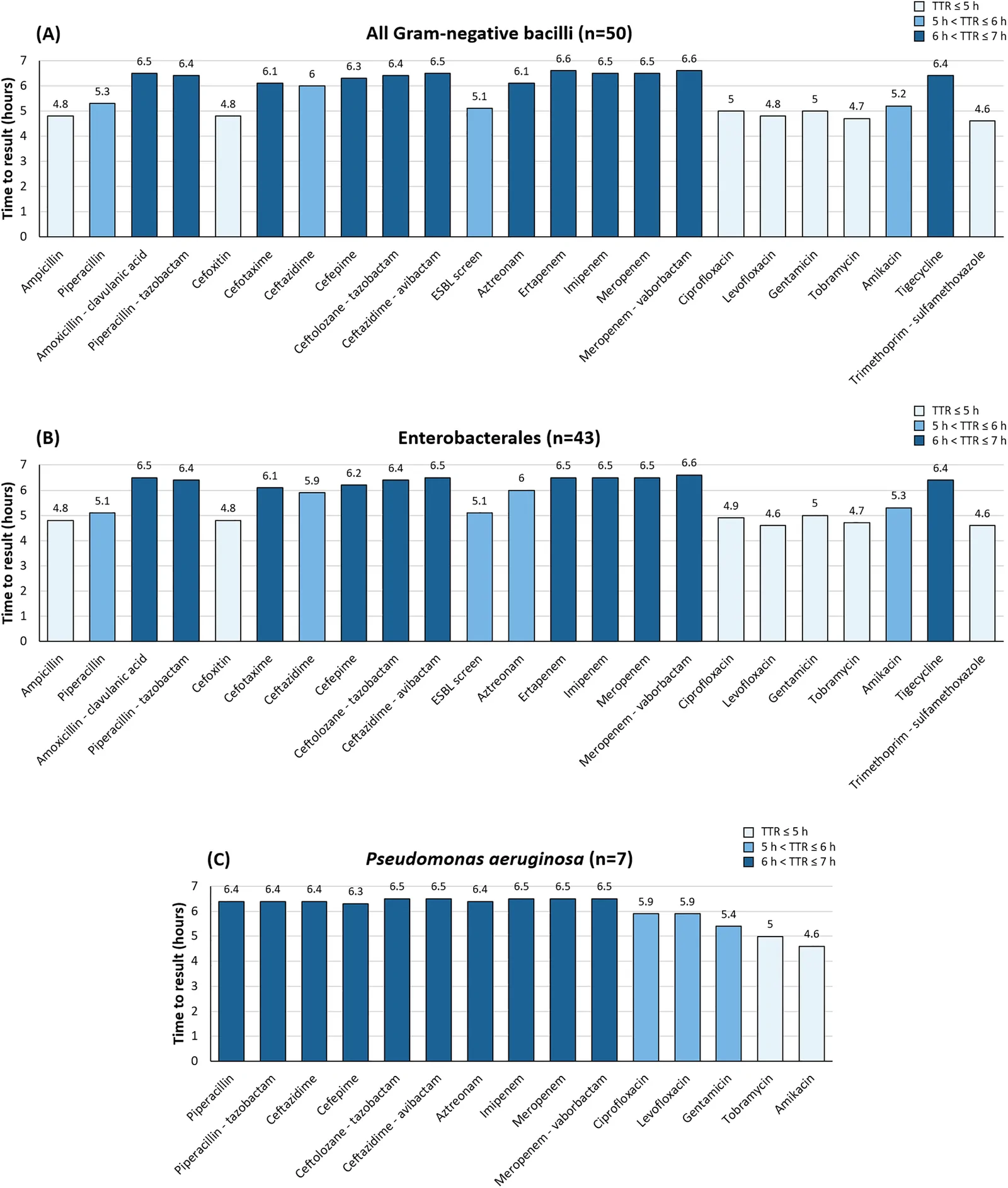
Average time to result for each antibiotic tested using the Vitek^®^ REVEAL^TM^ system. (A) All Gram-negative bacilli (n = 50); (B) Enterobacterales isolates (n = 43); (C) *Pseudomonas aeruginosa* isolates (n = 7).

The TTR for individual antibiotics using the Vitek^®^ REVEAL^TM^ system ranged from 3.2 h (e.g., ampicillin, ciprofloxacin and trimethoprim-sulfamethoxazole) to 8.5 h (meropenem-vaborbactam) (**S1 Fig**). Across the 985 strain-antibiotic combinations tested, the mean TTR was 5.7 h (Fig 2A). The median time to obtain a complete Vitek^®^ REVEAL^TM^ AST result was 6.5 h.

Among the 43 Enterobacterales isolates, the average TTR was also 5.7 h. The fastest results were obtained for trimethoprim-sulfamethoxazole and levofloxacin, both with a mean TTR of 4.6 h. In contrast, cefotaxime, piperacillin-tazobactam and carbapenems required longer incubation times, with mean TTRs of 6.1, 6.4 and 6.5 h, respectively (Fig 2B).

For the seven *P. aeruginosa* isolates, the average TTR was 6.1 h. The earliest results were reported for amikacin and tobramycin, with mean TTRs of 4.6 and 5.0 h, respectively. The latest results were observed for carbapenems, ceftazidime-avibactam and ceftolozane-tazobactam, both with a mean TTR of 6.5 h (Fig 2C).

### Clinical performance

A total of 791 valid strain-antibiotic combinations were analyzed to assess the clinical performance of the Vitek^®^ REVEAL^TM^ system. Three invalid results were recorded: one for *E. coli* (cefotaxime), one for *P. mirabilis* (cefotaxime and ceftazidime), and one for *P. aeruginosa* isolate, which required repeat testing. Enterobacterales isolates were excluded for ceftolozane-tazobactam and ceftazidime-avibactam, as these agents were not tested by routine methods. For amoxicillin-clavulanic acid, sixteen strains were excluded from analysis due to a software update (susceptibility not reported by Vitek^®^ REVEAL^TM^).

Of the 789 exploitable combinations, 771 results were concordant with standard AST, yielding an overall CA of 97.7%. Two Enterobacterales isolates with MICs in the area of technical uncertainty for piperacillin-tazobactam (16 mg/L) were excluded from CA and error rate calculations. All quality control strains produced expected results.

For Enterobacterales (705 strain-antibiotic combinations), the overall CA was 98% (**Table 4**). The CA exceeded 90% for all 18 antimicrobials tested. Six VME were observed: two for piperacillin-tazobactam, and one each for piperacillin, cefotaxime, tobramycin, and tigecycline. One *E. cloacae* isolate associated with two VMEs (for piperacillin-tazobactam and cefotaxime) showed low inoculum on disk diffusion, which may have contributed to the discrepancies. Eight MEs were recorded, involving ertapenem (n = 2), piperacillin (n = 2), gentamicin (n = 2), ampicillin (n = 1), and tigecycline (n = 1). The ESBL screen test correctly identified both ESBL-producing isolates (one SHV-2-producing *K. pneumoniae* and one SHV-102-producing *E. coli*). Among two *E. coli* isolates overproducing AmpC, the ESBL screen test was positive for one strain and indeterminate for the other one.

**Table 4:** Clinical performance of the Vitek^®^ REVEAL^TM^ system for antimicrobial susceptibility testing of Enterobacterales (n = 43) and *Pseudomonas aeruginosa* (n = 7) isolates, compared to standard methods.

Antibiotic (n. of strains)	Conventional AST			Vitek^®^ REVEAL^TM^ AST			CA		No. of errors		
	S	I	R	S	I	R	n/N	%	VME	ME	mE
**Enterobacterales**											
Ampicillin (32)	16	0	16	15	0	17	31/32	96.9	0	1	NA
Piperacillin (38)	19	0	19	17	0	21	35/38	92.1	1	2	NA
Amoxicillin – clavulanic acid (21)	16	0	5	16	0	5	21/21	100	0	0	NA
Piperacillin – tazobactam (40)	34	0	6	36	0	4	38/40	95	2	0	NA
Cefoxitin (37)	34	0	3	34	0	3	37/37	100	0	0	NA
Cefotaxime (41)	36	0	5	37	0	4	40/41	97.6	1	0	0
Ceftazidime (42)	36	0	6	36	0	6	42/42	100	0	0	0
Cefepime (42)	39	1	2	39	1	2	42/42	100	0	0	0
Aztreonam (41)	36	2	3	36	2	3	41/41	100	0	0	0
Ertapenem (43)	43	0	0	41	0	2	41/43	95.3	0	2	NA
Imipenem (41)	41	0	0	41	0	0	41/41	100	0	0	0
Meropenem (43)	43	0	0	43	0	0	43/43	100	0	0	0
Ciprofloxacin (43)	41	0	2	41	0	2	43/43	100	0	0	0
Gentamicin (43)	42	0	1	40	0	3	41/43	95.3	0	2	NA
Tobramycin (43)	42	0	1	43	0	0	42/43	97.7	1	0	NA
Amikacin (43)	43	0	0	43	0	0	43/43	100	0	0	NA
Tigecycline (29)	28	0	1	28	0	1	27/29	93.1	1	1	NA
Trimethoprim – sulfamethoxazole (43)	35	0	8	35	0	8	43/43	100	0	0	0
**Total**	624 (88.5%)	3 (0.4%)	78 (11.1%)	621 (88.1%)	3 (0.4%)	81 (11.5%)	691/705	98	6 (7.7%)	8 (1.3%)	0 (0%)
***Pseudomonas aeruginosa***											
Piperacillin (7)	0	6	1	0	7	0	6/7	85.7	1	0	0
Piperacillin – tazobactam (7)	0	6	1	0	7	0	6/7	85.7	1	0	0
Ceftazidime (7)	0	6	1	0	6	1	7/7	100	0	0	0
Cefepime (7)	0	6	1	0	6	1	7/7	100	0	0	0
Ceftolozane – tazobactam (7)	7	0	0	7	0	0	7/7	100	0	0	0
Ceftazidime – avibactam (7)	7	0	0	7	0	0	7/7	100	0	0	0
Aztreonam (7)	0	7	0	0	6	1	6/7	85.7	0	0	1
Imipenem (7)	0	6	1	0	6	1	7/7	100	0	0	0
Meropenem (7)	6	1	0	7	0	0	6/7	85.7	0	0	1
Ciprofloxacin (7)	0	7	0	0	7	0	7/7	100	0	0	0
Tobramycin (7)	7	0	0	7	0	0	7/7	100	0	0	0
Amikacin (7)	7	0	0	7	0	0	7/7	100	0	0	0
**Total**	34 (40.5%)	45 (53.6%)	5 (5.9%)	35 (41.7%)	45 (53.6%)	4 (4.8%)	80/84	95.2	2	0	2

S, Susceptible; I, susceptible Increased exposure; R, Resistant; AST, Antimicrobial Susceptibility Testing; CA, Categorical Agreement; VME, Very Major Error; ME, Major Error; mE, minor Error; NA, Not Applicable. Error rates were not calculated for *Pseudomonas aeruginosa* because the number of strains was less than 10.

For *P. aeruginosa* strains, the overall CA was 95.1% (**Table 4**). However, CA fell below 90% for four antimicrobials (piperacillin, piperacillin-tazobactam, aztreonam, and meropenem). One VME was observed for piperacillin and one for piperacillin-tazobactam. Minor errors were recorded for aztreonam and meropenem. These findings should be interpreted with caution due to the limited number of isolates.

## Discussion

Antimicrobial resistance and long turnaround times for pathogen identification and AST are major contributors to delays in initiating effective therapy, particularly in critically ill patients with sepsis or septic shock [12,13,24]. Rapid availability of AST results is therefore essential to guide timely and appropriate antimicrobial treatment [8,24].

In this study, we evaluated the analytical performance and clinical contribution of two rapid diagnostic tools, the BioFire^®^ BCID2 molecular test and the Vitek^®^ REVEAL^TM^ system, for the management of GNB-BSI in ICU patients. The BioFire^®^ BCID2 panel has demonstrated clinical utility in several studies [15,16,18]. It is one of the most comprehensive rapid molecular assays, targeting a broad range of pathogens and key resistance genes, including the globally prevalent ESBL-encoding gene, *bla*_CTX-M_, and major carbapenemase-encoding genes [18]. In our study, BioFire^®^ BCID2 showed full concordance with MALDI-TOF identification but did not detect any resistance gene among the 50 GNB isolates, despite the presence of eight multidrug-resistant (MDR) isolates. This discrepancy is explained by the fact that the resistance mechanisms involved are not included in the BCID2 panel: 2 ESBL (SHV-2 and SHV-102) and 6 overproduced chromosomal β-lactamases (OXY-1, AmpC). This highlights a key limitation of molecular panels: a negative result for resistance genes does not equate to a wild-type phenotype, as multiple resistance mechanisms may be undetectable by current assays. Another strength of the BCID2 panel is its ability to detect some polymicrobial infections (half of the cases in this study), which are not uncommon in ICU settings [18,25]. As previously noted by Berinson *et al.*, rapid AST methods such as Vitek^®^ REVEAL^TM^ are valuable complements to molecular diagnostics, providing actionable susceptibility data [16].

Our findings confirm the significant time saving offered by both tools. Compared to standard methods, BioFire^®^ BCID2 and Vitek^®^ REVEAL^TM^ reduced the median time to identification and AST by 19 h and 14.9 h, respectively, constituting a substantial benefit in the management of critically ill patients [24]. The Vitek^®^ REVEAL^TM^ system is particularly attractive due to its ease of use, ability to test last-resort antibiotics, and independence from prior species identification (although identification is required for result interpretation). The mean global TTR of 5.7 h observed in our study is consistent with previous reports [26–29]. For example, Couchot *et al.* reported a mean TTR of 6.4 h for *P. aeruginosa* in spiked BCs, which aligns with our findings for clinical *P. aeruginosa* isolates (6.1 h) [30]. Some studies have reported TTRs of ≤ 5 h [31–33]. It has been suggested that TTR may be shorter for resistant strains [27,29]. This could explain the slightly longer TTR in our cohort in which the resistance rate was relatively low. In any case, it is important to underline that a complete AST result (median of 6.5 h in our study) is essential in order to enable microbiological interpretation of the data, especially for the MDR strains.

Beyond speed, the Vitek^®^ REVEAL^TM^ system demonstrated excellent clinical performance. Overall CA exceeded the 90% threshold recommended by ISO 20776–2:2007 [23], consistent with previous studies [26–34]. For Enterobacterales, CA was above 90% for all tested antimicrobials. For *P. aeruginosa*, CA fell below 90% for four antibiotics, though this should be interpreted cautiously due to the small sample size.

Among the discrepancies, three VME involved piperacillin-tazobactam, an agent known for its challenging AST interpretation [35]. The affected isolates included an *E. cloacae* complex and a *P. aeruginosa* overproducing AmpC, and an *E. coli* with overproduced penicillinase. This is clinically relevant, as piperacillin-tazobactam is frequently used empirically to treat GNB infections in ICU settings [36]. In one case, a patient with an AmpC-overproducing *E. cloacae* complex received inappropriate monotherapy (piperacillin-tazobactam) based on a false-susceptible Vitek^®^ REVEAL^TM^ result, with therapy adjusted only after the conventional AST, approximately 14 h later. Notably, this isolate also showed a false-susceptible result for cefotaxime, and the disk diffusion plate performed directly on the positive BC showed low inoculum, suggesting reduced VOC production, which may have contributed to the two VMEs. However, Tibbets *et al.* showed that there was no correlation between colony forming units count and AST accuracy (ref 29), and Ostermann *et al.* found no correlation between bacterial count at the time of AST initiation and error rates using flow cytometry [29].

The ESBL screen test included in the Vitek^®^ REVEAL^TM^ panel successfully detected both ESBL-producing strains but also yielded a false-positive result for an AmpC-overproducing *E. coli*. This lack of specificity has not been widely reported, though differences in antimicrobial panels and test configurations across studies may account for this [26–29,31–34].

Other discrepancies involved carbapenems, though none resulted in VMEs. One Extended-spectrum AmpC (ESAC)-producing *E. cloacae* complex and one ESBL-producing *K. pneumoniae* were falsely resistant to ertapenem by Vitek^®^ REVEAL^TM^, while one *P. aeruginosa* with OprD deficiency was falsely susceptible to meropenem, despite being categorized as susceptible with increased exposure by standard methods.

Finally, our findings underscore the importance of optimizing pre-analytical steps to further reduce turnaround times. In particular, the delay between sample collection and BC incubation was non-negligible. Furthermore, emerging technologies, such as blood culture-free ultra-rapid AST platforms that operate directly on whole blood, may offer future solutions bypassing the incubation step altogether [37]. Further validation of such approaches is needed to assess their clinical impact.

This study has several limitations. First, the evaluation of the Vitek^®^ REVEAL^TM^ system was conducted on a relatively small number of GNB isolates, with *E. coli* representing 60% of the cohort. The overall prevalence of antimicrobial resistance was low, and no carbapenem-resistant Enterobacterales were included. Additionally, Vitek^®^ REVEAL^TM^ results were primarily compared to disk diffusion testing. While this method is widely accepted and reliable for many antibiotics, it does not allow for the calculation of essential agreement or bias, as recommended by ISO 20776–2:2021 [38]. Operational constraints during the study period also impacted turnaround times. BCs that flagged positive between midnight and 8:00 AM were not processed until the morning shift, leading to delays for 21 patients. Since then, our laboratory has transitioned to 24/7 operations, which is expected to significantly reduce these delays in future practice.

Some limitations of the Vitek^®^ REVEAL^TM^ system itself should also be noted. Although the validated GNB species cover the most common pathogens involved in BSI [2,4], the inability to test additional clinically relevant species, such as *Serratia marcescens*, which accounted for five BSI episodes during the study period, limits its applicability. Furthermore, the ESBL screen test is not reported for group III Enterobacterales, and cefepime susceptibility is not provided for *K. aerogenes*. This is a notable omission, as cefepime is a key agent for carbapenem-sparing strategies in the context of AmpC overproduction [39]. Finally, an open access expert system software would be beneficial to better align with local recommendations of AST results interpretation.

In conclusion, the combination of the BioFire^®^ BCID2 panel and the Vitek^®^ REVEAL^TM^ system significantly reduced turnaround times for both pathogen identification and AST under real-life ICU conditions for patients with GNB-BSI. These rapid diagnostic tools demonstrated strong analytical performance and clinical relevance. To maximize their impact, their implementation should be embedded within an optimized laboratory workflow and closely integrated with antimicrobial stewardship programs. Such integration is essential to translate diagnostic speed into improved patient outcomes and to support the global effort to preserve antimicrobials efficacy [40].

## Supporting information

S1 Fig Range of time to result by antibiotic for all Gram-negative bacilli tested with the Vitek^®^ REVEAL^TM^ system. (PDF)

S1 Table List of antimicrobials included in the Vitek^®^ REVEAL^TM^ GN01 panel used for rapid AST. (PDF)

S2 Table Characteristics of bloodstream infection episodes excluded from the study during the inclusion period. (PDF)

S3 Table Details of bloodstream infections cases involving species not included in the Vitek^®^ REVEAL^TM^ panel. (PDF)

## References

[1] Goto M, Al-Hasan MN. Overall burden of bloodstream infection and nosocomial bloodstream infection in North America and Europe. Clin Microbiol Infect. 2013;19(6):501–9. doi: 10.1111/1469-0691.12195 23473333

[2] Kern WV, Rieg S. Burden of bacterial bloodstream infection-a brief update on epidemiology and significance of multidrug-resistant pathogens. Clin Microbiol Infect. 2020;26(2):151–7. doi: 10.1016/j.cmi.2019.10.031 31712069

[3] Mun SJ, Kim S-H, Kim H-T, Moon C, Wi YM. The epidemiology of bloodstream infection contributing to mortality: the difference between community-acquired, healthcare-associated, and hospital-acquired infections. BMC Infect Dis. 2022;22(1):336. doi: 10.1186/s12879-022-07267-9 35382769 PMC8981700

[4] Diekema DJ, Hsueh P-R, Mendes RE, Pfaller MA, Rolston KV, Sader HS, et al. The Microbiology of Bloodstream Infection: 20-Year Trends from the SENTRY Antimicrobial Surveillance Program. Antimicrob Agents Chemother. 2019;63(7):e00355-19. doi: 10.1128/AAC.00355-19 31010862 PMC6591610

[5] Vincent J-L, Sakr Y, Singer M, Martin-Loeches I, Machado FR, Marshall JC, et al. Prevalence and Outcomes of Infection Among Patients in Intensive Care Units in 2017. JAMA. 2020;323(15):1478–87. doi: 10.1001/jama.2020.2717 32207816 PMC7093816

[6] Calvo M, Stefani S, Migliorisi G. Bacterial Infections in Intensive Care Units: Epidemiological and Microbiological Aspects. Antibiotics (Basel). 2024;13(3):238. doi: 10.3390/antibiotics13030238 38534673 PMC10967584

[7] Zasowski EJ, Bassetti M, Blasi F, Goossens H, Rello J, Sotgiu G, et al. A Systematic Review of the Effect of Delayed Appropriate Antibiotic Treatment on the Outcomes of Patients With Severe Bacterial Infections. Chest. 2020;158(3):929–38. doi: 10.1016/j.chest.2020.03.087 32446623

[8] Van Heuverswyn J, Valik JK, Desirée van der Werff S, Hedberg P, Giske C, Nauclér P. Association Between Time to Appropriate Antimicrobial Treatment and 30-day Mortality in Patients With Bloodstream Infections: A Retrospective Cohort Study. Clin Infect Dis. 2023;76(3):469–78. doi: 10.1093/cid/ciac727 36065752 PMC9907509

[9] Antimicrobial Resistance Collaborators. Global burden of bacterial antimicrobial resistance in 2019: a systematic analysis. Lancet. 2022;399(10325):629–55. doi: 10.1016/S0140-6736(21)02724-0 35065702 PMC8841637

[10] European Centre for Disease Prevention and Control. Antimicrobial resistance in the EU/EEA (EARS-Net) - Annual epidemiological report for 2022. Stockholm: ECDC. 2023.

[11] World Health Organization. Global antimicrobial resistance and use surveillance system (GLASS) report: 2022. Geneva: WHO. 2022.

[12] Battle SE, Bookstaver PB, Justo JA, Kohn J, Albrecht H, Al-Hasan MN. Association between inappropriate empirical antimicrobial therapy and hospital length of stay in Gram-negative bloodstream infections: stratification by prognosis. J Antimicrob Chemother. 2017;72(1):299–304. doi: 10.1093/jac/dkw402 27986899

[13] Kadri SS, Lai YL, Warner S, Strich JR, Babiker A, Ricotta EE, et al. Inappropriate empirical antibiotic therapy for bloodstream infections based on discordant in-vitro susceptibilities: a retrospective cohort analysis of prevalence, predictors, and mortality risk in US hospitals. Lancet Infect Dis. 2021;21(2):241–51. doi: 10.1016/S1473-3099(20)30477-1 32916100 PMC7855478

[14] Timbrook TT, Morton JB, McConeghy KW, Caffrey AR, Mylonakis E, LaPlante KL. The Effect of Molecular Rapid Diagnostic Testing on Clinical Outcomes in Bloodstream Infections: A Systematic Review and Meta-analysis. Clin Infect Dis. 2017;64(1):15–23. doi: 10.1093/cid/ciw649 27678085

[15] Verroken A, Despas N, Rodriguez-Villalobos H, Laterre P-F. The impact of a rapid molecular identification test on positive blood cultures from critically ill with bacteremia: A pre-post intervention study. PLoS One. 2019;14(9):e0223122. doi: 10.1371/journal.pone.0223122 31557233 PMC6762135

[16] Berinson B, Both A, Berneking L, Christner M, Lütgehetmann M, Aepfelbacher M, et al. Usefulness of BioFire FilmArray BCID2 for Blood Culture Processing in Clinical Practice. J Clin Microbiol. 2021;59(8):e0054321. doi: 10.1128/JCM.00543-21 33980648 PMC8373244

[17] Rhoads DD, Pournaras S, Leber A, Balada-Llasat J-M, Harrington A, Sambri V, et al. Multicenter Evaluation of the BIOFIRE Blood Culture Identification 2 Panel for Detection of Bacteria, Yeasts, and Antimicrobial Resistance Genes in Positive Blood Culture Samples. J Clin Microbiol. 2023;61(6):e0189122. doi: 10.1128/jcm.01891-22 37227281 PMC10281132

[18] Hattab S, Ma AH, Tariq Z, Vega Prado I, Drobish I, Lee R, et al. Rapid Phenotypic and Genotypic Antimicrobial Susceptibility Testing Approaches for Use in the Clinical Laboratory. Antibiotics (Basel). 2024;13(8):786. doi: 10.3390/antibiotics13080786 39200086 PMC11351821

[19] Weisskopf L, Schulz S, Garbeva P. Microbial volatile organic compounds in intra-kingdom and inter-kingdom interactions. Nat Rev Microbiol. 2021;19(6):391–404. doi: 10.1038/s41579-020-00508-1 33526910

[20] Oudiane L, Benyahia M, Salipante F, Dubois A, Muller L, Lavigne J-P, et al. Clinical impact of the BCID2 and rapid AST VITEK® REVEALTM on antibiotic optimisation in critically ill patients with Gram-negative bloodstream infections: a quasi-experimental pre/post interventional study. J Antimicrob Chemother. 2025;80(10):2665–75. doi: 10.1093/jac/dkaf271 40754712

[21] Société Française de Microbiologie. Recommandations 2023 Version 1.0. Comité de l’Antibiogramme de la Société Française de Microbiologie. 2023.

[22] European Committee on Antimicrobial Susceptibility Testing. Clinical breakpoints and dosing of antibiotics. 2023.

[23] Clinical laboratory testing and in vitro diagnostic test systems - Susceptibility testing of infectious agents and evaluation of performance of antimicrobial susceptibility test devices - Part 2: Evaluation of performance of antimicrobial susceptibility test devices. ISO 20776-2:2007. iTeh Standards. https://standards.iteh.ai/catalog/standards/cen/e58114a5-4af0-488d-addf-f410a8445ee0/en-iso-20776-2-2007

[24] Kollef MH, Shorr AF, Bassetti M, Timsit J-F, Micek ST, Michelson AP, et al. Timing of antibiotic therapy in the ICU. Crit Care. 2021;25(1):360. doi: 10.1186/s13054-021-03787-z 34654462 PMC8518273

[25] Banerjee R, Patel R. Molecular diagnostics for genotypic detection of antibiotic resistance: current landscape and future directions. JAC Antimicrob Resist. 2023;5(1):dlad018. doi: 10.1093/jacamr/dlad018 36816746 PMC9937039

[26] Antonelli A, Cuffari S, Casciato B, Giani T, Rossolini GM. Evaluation of the Vitek® Reveal™ system for rapid antimicrobial susceptibility testing of Gram-negative pathogens, including ESBL, CRE and CRAB, from positive blood cultures. Diagn Microbiol Infect Dis. 2024;110(4):116503. doi: 10.1016/j.diagmicrobio.2024.116503 39197326

[27] Menchinelli G, Squitieri D, Magrì C, De Maio F, D’Inzeo T, Cacaci M, et al. Verification of the Vitek Reveal System for Direct Antimicrobial Susceptibility Testing in Gram-Negative Positive Blood Cultures. Antibiotics (Basel). 2024;13(11):1058. doi: 10.3390/antibiotics13111058 39596752 PMC11590937

[28] Bonetti C, Radaelli GM, De Francesco MA. Susceptibility of Gram-negative bacteria from blood cultures assessed by a rapid antimicrobial susceptibility assay, the VITEK® Reveal™: a preliminary study. J Med Microbiol. 2024;73(12):10.1099/jmm.0.001941. doi: 10.1099/jmm.0.001941 39629793

[29] Ostermann G, Körber-Irrgang B, Krüger A, Singh P, Vo K, Gielen J, et al. Performance evaluation of the Specific Reveal system for rapid antibiotic susceptibility testing from positive blood cultures containing Gram-negative pathogens. J Clin Microbiol. 2024;62(12):e0069224. doi: 10.1128/jcm.00692-24 39545740 PMC11633213

[30] Couchot J, Fournier D, Bour M, Rajeev L, Rhodes P, Singh P, et al. Evaluation of the Reveal® rapid AST system to assess the susceptibility of Pseudomonas aeruginosa from blood cultures. Eur J Clin Microbiol Infect Dis. 2023;42(3):359–63. doi: 10.1007/s10096-023-04556-2 36729319

[31] Tibbetts R, George S, Burwell R, Rajeev L, Rhodes PA, Singh P, et al. Performance of the Reveal Rapid Antibiotic Susceptibility Testing System on Gram-Negative Blood Cultures at a Large Urban Hospital. J Clin Microbiol. 2022;60(6):e0009822. doi: 10.1128/jcm.00098-22 35607972 PMC9199398

[32] Bianco G, Boattini M, Comini S, Bondi A, Curtoni A, Piccinini G, et al. Detection of volatile organic compounds as new paradigm to accelerate antimicrobial susceptibility testing: performance evaluation of VITEK® REVEAL™. J Antimicrob Chemother. 2024;79(9):2237–45. doi: 10.1093/jac/dkae219 38958300

[33] Girlich D, Jousset AB, Emeraud C, Rezzoug I, Burwell R, Singh P, et al. Evaluation of the Reveal® AST (SPECIFIC) for Antimicrobial Susceptibility Testing from Positive Blood Culture Spiked with Carbapenem-Resistant Isolates. Pathogens. 2024;13(9):722. doi: 10.3390/pathogens13090722 39338914 PMC11434930

[34] Calvo M, Maugeri G, Migliorisi G, Scalia G, Stefani S. The volatile organic compounds detection in MDR Gram-negatives antimicrobial susceptibility testing: Results from a four-month laboratory experience. Diagn Microbiol Infect Dis. 2024;110(4):116533. doi: 10.1016/j.diagmicrobio.2024.116533 39270517

[35] Henderson A, Humphries R. Building a Better Test for Piperacillin-Tazobactam Susceptibility Testing: Would that It Were So Simple (It’s Complicated). J Clin Microbiol. 2020;58(2):e01649-19. doi: 10.1128/JCM.01649-19 31723014 PMC6989057

[36] Tabah A, Lipman J, Barbier F, Buetti N, Timsit JF. Use of antimicrobials for bloodstream infections in the intensive care unit, a clinically oriented review. Antibiotics. 2022;11:362. doi: 10.3390/antibiotics11030362 35326825 PMC8944491

[37] Kim TH, Kang J, Jang H, Joo H, Lee GY, Kim H, et al. Blood culture-free ultra-rapid antimicrobial susceptibility testing. Nature. 2024;632(8026):893–902. doi: 10.1038/s41586-024-07725-1 39048820

[38] ISO. EN ISO 20776-2:2021 - Clinical laboratory testing and in vitro diagnostic test systems - Susceptibility testing of infectious agents and evaluation of performance of antimicrobial susceptibility test devices - Part 2: Evaluation of performance of antimicrobial susceptibility test devices against reference broth micro-dilution. iTeh Standards. https://standards.iteh.ai/catalog/standards/cen/92fc193a-5135-4d74-b3b5-a6962a16e505/en-iso-20776-2-2022

[39] Herrmann J, Burgener-Gasser A-V, Goldenberger D, Roth J, Weisser M, Tamma PD, et al. Cefepime versus carbapenems for treatment of AmpC beta-lactamase-producing Enterobacterales bloodstream infections. Eur J Clin Microbiol Infect Dis. 2024;43(2):213–21. doi: 10.1007/s10096-023-04715-5 37993680 PMC10821988

[40] Peri AM, Chatfield MD, Ling W, Furuya-Kanamori L, Harris PNA, Paterson DL. Rapid Diagnostic Tests and Antimicrobial Stewardship Programs for the Management of Bloodstream Infection: What Is Their Relative Contribution to Improving Clinical Outcomes? A Systematic Review and Network Meta-analysis. Clin Infect Dis. 2024;79(2):502–15. doi: 10.1093/cid/ciae234 38676943 PMC11327801

