# Supplementary material for: Real-life evaluation of BioFire® BCID2 and Vitek® REVEALTM for Gram-negative bloodstream infections in critical care
Source: PLoS One. 2026 Sep 10;21(9):e0356199. doi: 10.1371/journal.pone.0356199 (PMC13561357; doi:10.1371/journal.pone.0356199)

S1 Fig. Range of time to result by antibiotic for all Gram-negative bacilli tested with the Vitek® REVEAL™ system.

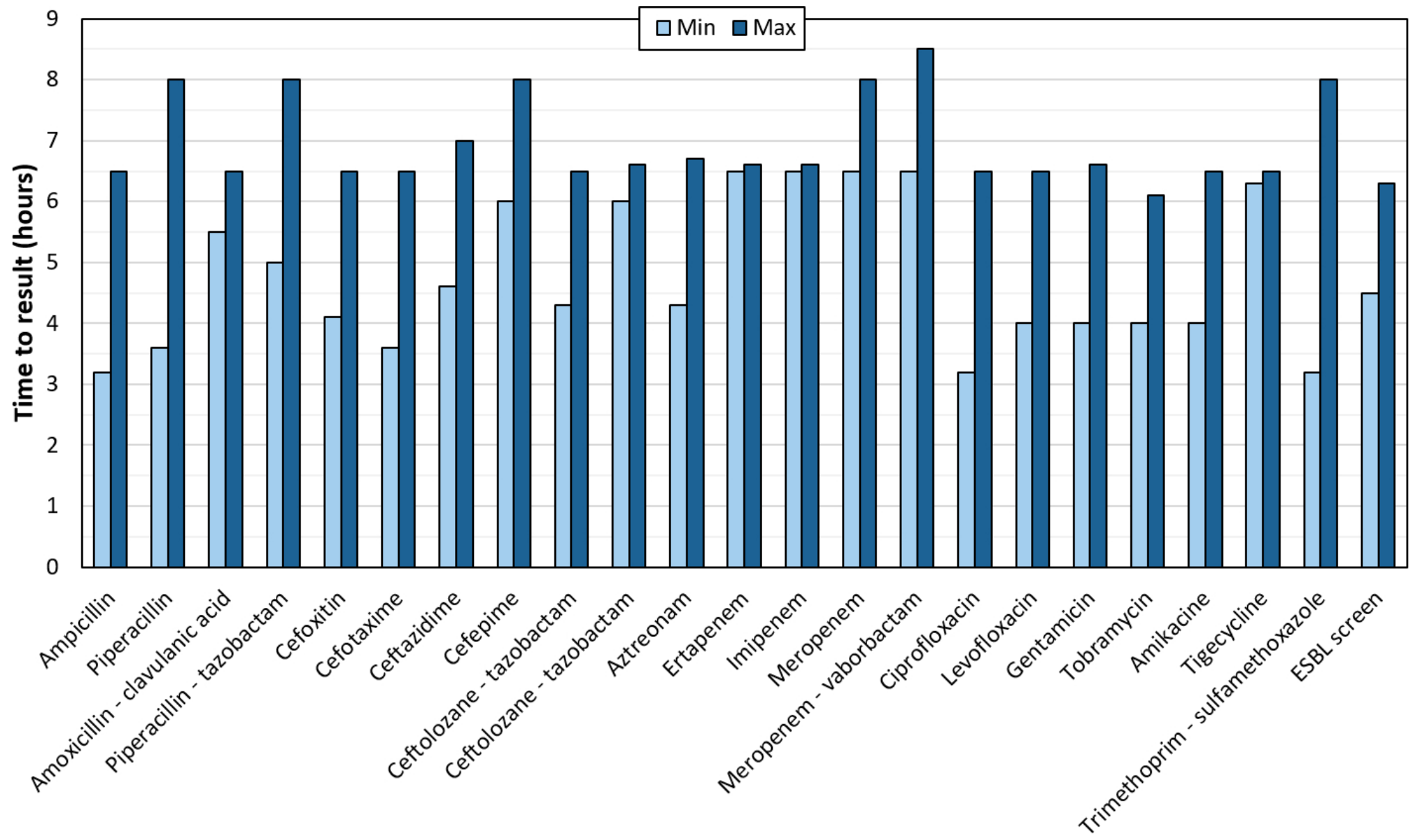

Supplement: Supplementary file 1 [file pone.0356199.s001.pdf]
