# Supplementary material for: Real-life evaluation of BioFire® BCID2 and Vitek® REVEALTM for Gram-negative bloodstream infections in critical care
Source: PLoS One. 2026 Sep 10;21(9):e0356199. doi: 10.1371/journal.pone.0356199 (PMC13561357; doi:10.1371/journal.pone.0356199)
Supplement: Supplementary file 2 [file pone.0356199.s002.pdf]

S1 Table. List of antimicrobials included in the Vitek® REVEAL™ GN01 panel used for rapid AST.

β-lactams		Other antimicrobials
Ampicillin	Ceftolozane - tazobactam	Ciprofloxacin
Piperacillin	Ceftazidime - avibactam	Levofloxacin
Amoxicillin - clavulanic acid	Ceftazidime - clavulanic acid	Gentamicin
Piperacillin - tazobactam	Aztreonam	Tobramycin
Cefoxitin	Ertapenem	Amikacin
Cefotaxime	Imipenem	Tigecycline
Ceftazidime	Meropenem	Trimethoprim - sulfamethoxazole
Cefepime	Meropenem - vaborbactam	
