# Supplementary material for: Real-life evaluation of BioFire® BCID2 and Vitek® REVEALTM for Gram-negative bloodstream infections in critical care
Source: PLoS One. 2026 Sep 10;21(9):e0356199. doi: 10.1371/journal.pone.0356199 (PMC13561357; doi:10.1371/journal.pone.0356199)
Supplement: Supplementary file 3 [file pone.0356199.s003.pdf]

S2 Table. Characteristics of bloodstream infection episodes excluded from the study during the inclusion period.

Cause of non-inclusion	Number of bloodstream infections
Species out-of-panel	18
Polymicrobial blood culture	12
Technical incident	5
Moribund patient	4
Inclusion in other studies	2
Under legal protection	2
Refusal	1
**Total**	44
