# Supplementary material for: Real-life evaluation of BioFire® BCID2 and Vitek® REVEALTM for Gram-negative bloodstream infections in critical care
Source: PLoS One. 2026 Sep 10;21(9):e0356199. doi: 10.1371/journal.pone.0356199 (PMC13561357; doi:10.1371/journal.pone.0356199)
Supplement: Supplementary file 4 [file pone.0356199.s004.pdf]

S3 Table. Details of bloodstream infections cases caused by species not included in the Vitek® REVEAL™ panel.

Species out-of-panel	Number of blood cultures
Serratia marcescens	5
Bacteroides thetaiotaomicron	3
Haemophilus influenzae	2
Raoultella ornithinolytica	2
Salmonella enterica	1
Stenotrophomonas maltophilia	1
Fusobacterium necrophorum	1
Pseudomonas oryzihabitans	1
Acinetobacter ursingii	1
Aggregatibacter aprophilus	1
**Total**	18
